# Allicin and Capsaicin Ameliorated Hypercholesterolemia by Upregulating LDLR and Downregulating PCSK9 Expression in HepG2 Cells

**DOI:** 10.3390/ijms232214299

**Published:** 2022-11-18

**Authors:** Nantiya Nawaka, Smith Wanmasae, Arthit Makarasen, Decha Dechtrirat, Supanna Techasakul, Nutjaree Jeenduang

**Affiliations:** 1 School of Allied Health Sciences, Walailak University, Nakhon Si Thammarat 80160, Thailand; 2 Laboratory of Organic Synthesis, Chulabhorn Research Institute, Bangkok 10210, Thailand; 3 Department of Materials Science, Faculty of Science, Kasetsart University, Bangkok 10900, Thailand; 4 Specialized Center of Rubber and Polymer Materials for Agriculture and Industry (RPM), Faculty of Science, Kasetsart University, Bangkok 10900, Thailand; 5 Food Technology and Innovation Research Center of Excellence, Walailak University, Nakhon Si Thammarat 80160, Thailand

**Keywords:** allicin, capsaicin, hypercholesterolemia, LDLR, PCSK9

## Abstract

Hypercholesterolemia is a common cause of cardiovascular diseases (CVDs). Although allicin and capsaicin possess hypolipidemic effects through several molecular mechanisms, their effects on LDLR and PCSK9 expression are still unknown. This study aimed to investigate the effects of allicin and capsaicin on LDLR and PCSK9 expression in HepG2 cells. The effects of allicin and capsaicin on cell viability were evaluated by MTT assay and trypan blue exclusion assay. Low-density lipoprotein receptor (LDLR) levels and LDL uptake were determined by flow cytometry and confocal laser scanning microscopy (CLSM), respectively. RT-qPCR and Western blot analyses were performed to evaluate the expression of PCSK9, LDLR, SREBP-2, and HNF1α. ELISA was used to measure PCSK9 levels in culture media. Allicin and capsaicin increased the protein expression levels of LDLR via activation of the transcription factor SREBP2. However, allicin and capsaicin decreased the expression of PCSK9 protein and the secretion of PCSK9 in culture media via the suppression of HNF1α. Moreover, allicin and capsaicin increased LDL uptake into HepG2 cells. The efficacies of the hypolipidemic effects of allicin (200 µM) and capsaicin (200 µM) were comparable to that of atorvastatin (10 µM) in this study. In conclusion, allicin and capsaicin possessed hypolipidemic effects via the upregulation of LDLR and downregulation of PCSK9 expression, thereby enhancing LDL uptake into HepG2 cells. This indicates that allicin and capsaicin should be used as potent supplements to ameliorate hypercholesterolemia.

## 1. Introduction

Cardiovascular diseases (CVDs) are the leading cause of mortality in people worldwide [1]. CVDs result from several risk factors including hypercholesterolemia, hypertension, diabetes, obesity, and metabolic syndrome [2]. The most common CVD risk factor is hypercholesterolemia, which results from both environmental factors, e.g., smoking, diet, alcohol consumption and lack of exercise, and genetic factors [3,4]. Several medicinal plants have been reported to reduce serum lipids and further prevent CVDs [5].

Garlic (*Allium sativum* L.) is widely used in food, spice, and traditional medicines worldwide [6]. Previous studies have shown that garlic can be used to treat infections, rheumatism, heart disease, diabetes, hypertension, and hypercholesterolemia and to prevent atherosclerosis and cancer [6]. Garlic possesses antihypertensive, antibacterial, hypolipidemic, and hypoglycemic properties [7,8,9,10]. Garlic contains several organosulfur constituents such as S-allyl-cysteine sulfoxide (alliin), diallyl thiosulfonate (allicin), diallyl trisulfide (DATS), diallyl disulfide (DADS), diallyl sulfide (DAS), E/Z-ajoene, and S-allyl-cysteine (SAC) [11]. Allicin, a major active compound of garlic, is produced from alliin by alliinase, which is released and activated from chopped or crushed raw garlic [12].

*Capsicum annuum* (family: Solanaceae) is a pungent spice known as chili pepper or red pepper. *Capsicum annuum* consists of capsaicinoid compounds such as capsaicin, dihydrocapsaicin, homocapsaicin, and homodihydrocapsaicin [11,13]. The major component of capsaicinoid is capsaicin (trans-8-methyl-N-vanillyl-6-nonenamide), which has antioxidant, anti-inflammatory, weight-reducing, and hypolipidemic effects [14].

Proprotein convertase subtilisin/kexin type 9 (PCSK9) plays an important role in cholesterol homeostasis by binding to low-density lipoprotein receptor (LDLR) and then promoting its degradation in the lysosome [15,16]. This results in decreased LDLR, decreased LDL removal, and subsequently increased LDL-C in circulation [17,18]. Moreover, PCSK9 also plays a role in developing atherosclerosis via noncholesterol-related processes, e.g., inflammation, triglyceride (TG)-rich lipoprotein metabolism, and platelet activation [19]

Although allicin and capsaicin and their hypolipidemic effects have been studied, the molecular mechanisms of the expression of LDLR and PCSK9 in lipoprotein metabolism by these active compounds have not been elucidated. Consequently, the present study aimed to investigate the effects of allicin and capsaicin on the expression of LDLR and PCSK9 in HepG2 cells.

## 2. Results

### 2.1. Effects of Allicin and Capsaicin on the Viability of HepG2 Cells

**Figure 1:**
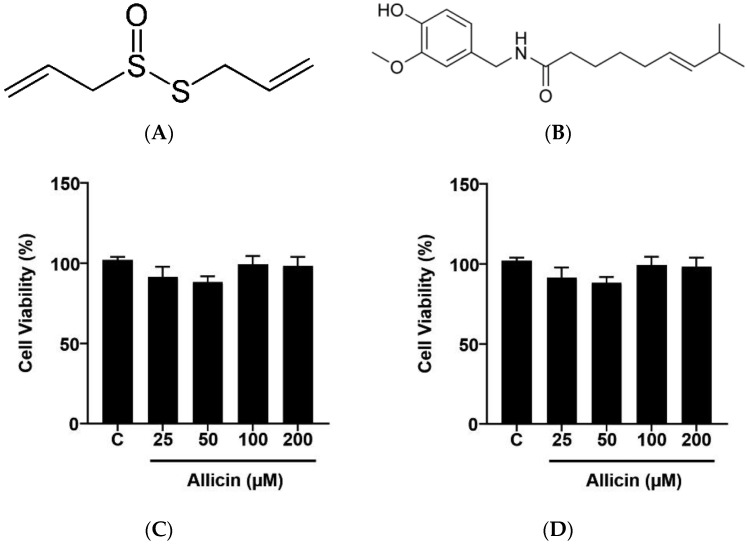

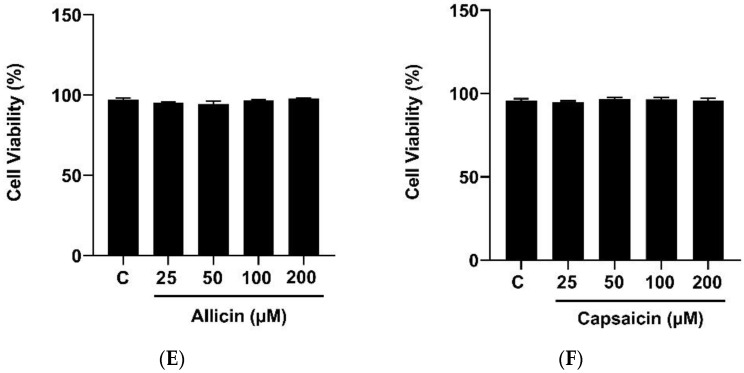
Effects of allicin and capsaicin on HepG2 cell viability. Chemical structure of allicin (**A**). Chemical structure of capsaicin (**B**). HepG2 cells were cultured in starvation medium and treated with vehicle (0.1% DMSO) or allicin (0, 25, 50, 100, 200 μM) (**C**,**E**) and capsaicin (0, 25, 50, 100, 200 μM) (**D**,**F**) for 24 h. Cell viability was measured using the MTT assay (**C**,**D**) and the trypan blue exclusion assay (**E**,**F**). The data represent the mean ± SD of three independent experiments (*N* = 3).

To investigate the effects of allicin and capsaicin on the cytotoxic effects on HepG2 cells, cells were treated with vehicle (0.1% DMSO), allicin (25, 50, 100, and 200 µM), or capsaicin (25, 50, 100, and 200 µM) for 24 h in DMEM, and cell viability was measured using the MTT assay and trypan blue dye exclusion. Treatment with allicin or capsaicin had no cytotoxic effect on HepG2 cells in either assay (Figure 1).

### 2.2. Effects of Allicin and Capsaicin on LDLR and PCSK9 Expression and LDL Uptake in HepG2 Cells

**Figure 2:**
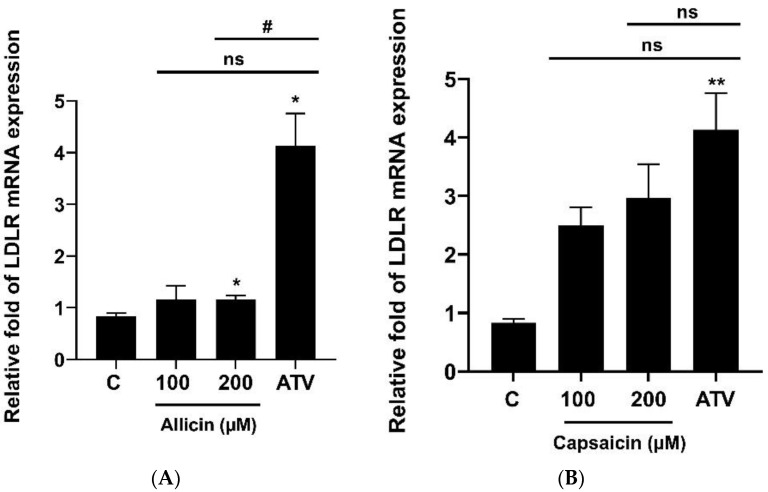

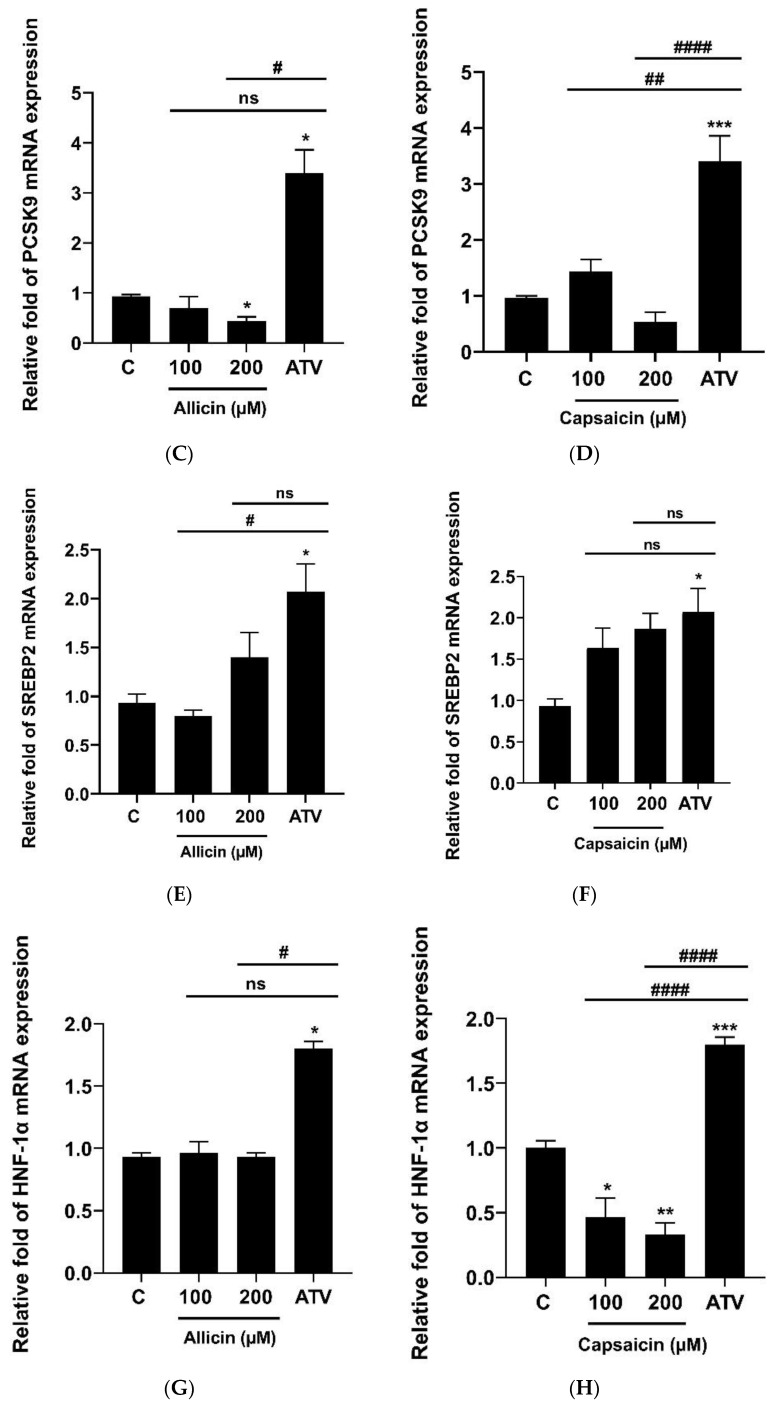
Effects of allicin (100, 200 µM) and capsaicin (100, 200 µM) on *LDLR* (**A**,**B**), *PCSK9* (**C**,**D**), *SREBP2* (**E**,**F**), and *HNF1α* (**G**,**H**) mRNA expression in HepG2 cells. (**A**) HepG2 cells were treated with allicin (100, 200 µM), capsaicin (100, 200 µM), or atorvastatin (10 µM) for 24 h. The mRNA expression of *LDLR* was determined by RT-qPCR. The data represent the mean ± SEM of three independent experiments (*N* = 3). * *p* < 0.05, ** *p* < 0.01, and *** *p* < 0.005 represent significant differences compared to the vehicle-treated cells or the control group (C). ^#^ *p* <0.05, ^##^ *p* < 0.01, and ^####^ *p* < 0.001 represent significant differences compared to atorvastatin (ATV)-treated cells. ns: not significant.

**Figure 3:**
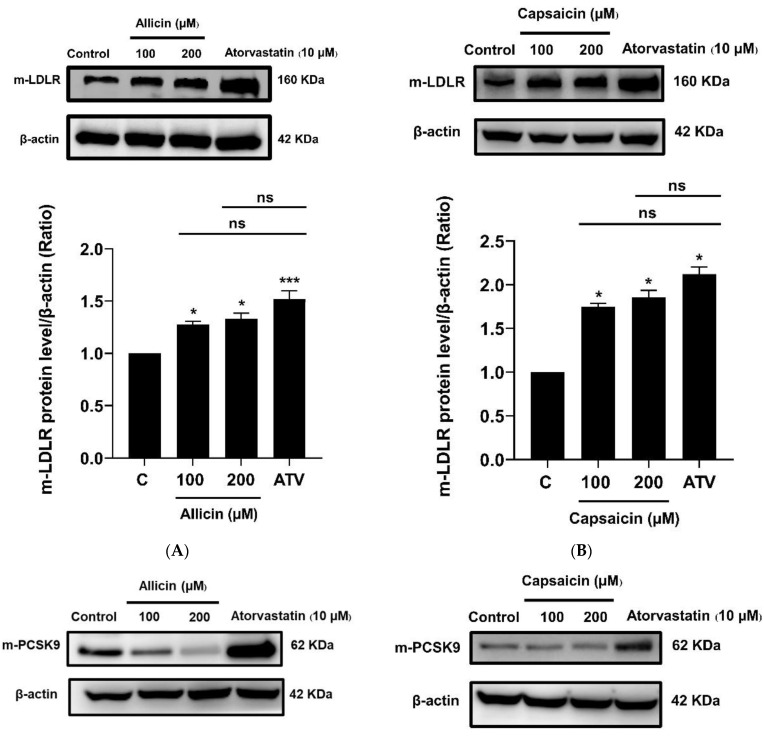

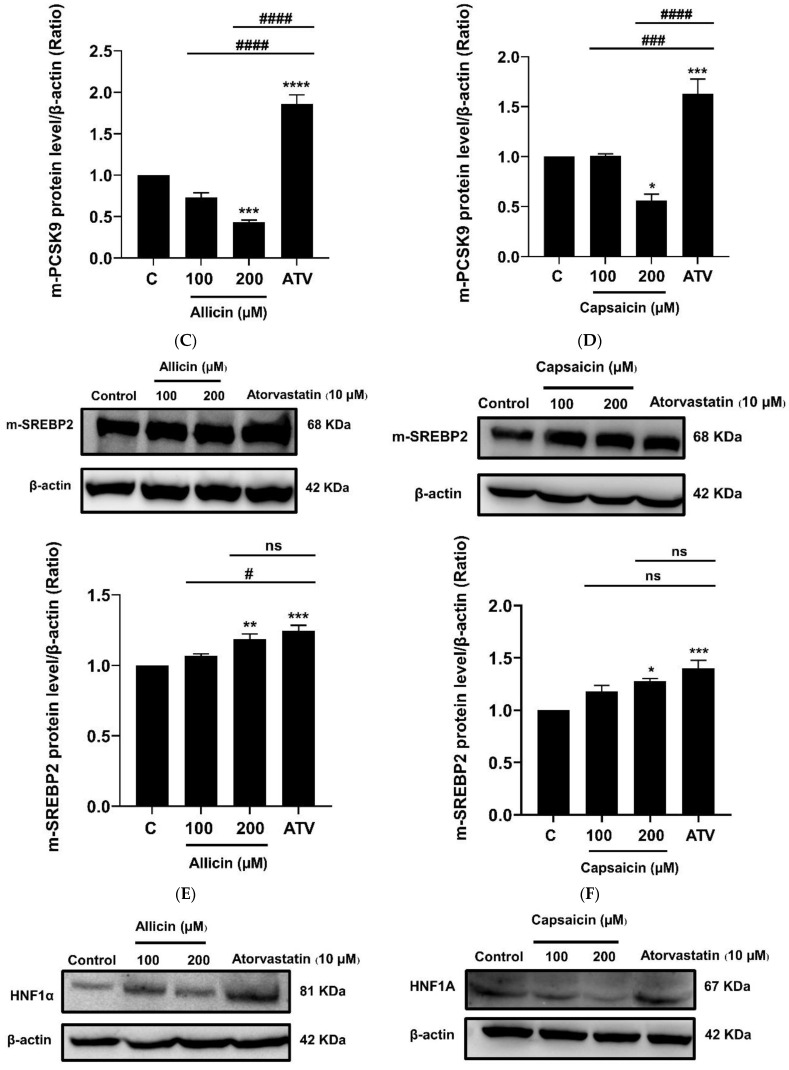

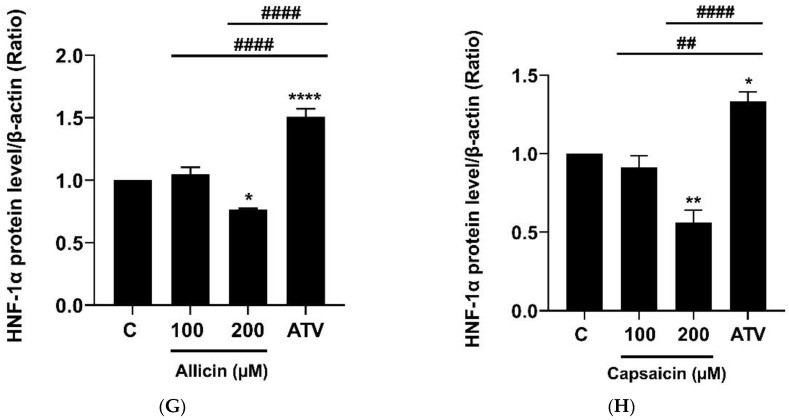
Effects of allicin (100, 200 µM) and capsaicin (100, 200 µM) on LDLR (**A**,**B**), PCSK9 (**C**,**D**), SREBP2 (**E**,**F**), and HNF1α (**G**,**H**) protein expression in HepG2 cells. The protein level of LDLR was determined by Western blot analysis. A representative blot is shown. The normalized intensity of LDLR versus β-actin and all data represent the mean ± SEM of three independent experiments (*N* = 3). * *p* < 0.05, ** *p* < 0.01, *** *p* < 0.005, and **** *p* < 0.001 represent significant differences compared to the vehicle-treated cells or the control group (C). ^#^ *p* < 0.05, ^##^ *p* < 0.01, ^###^ *p* < 0.005, and ^####^ *p* < 0.001 represent significant differences compared to atorvastatin (ATV)-treated cells. ns: not significant.

**Figure 4:**
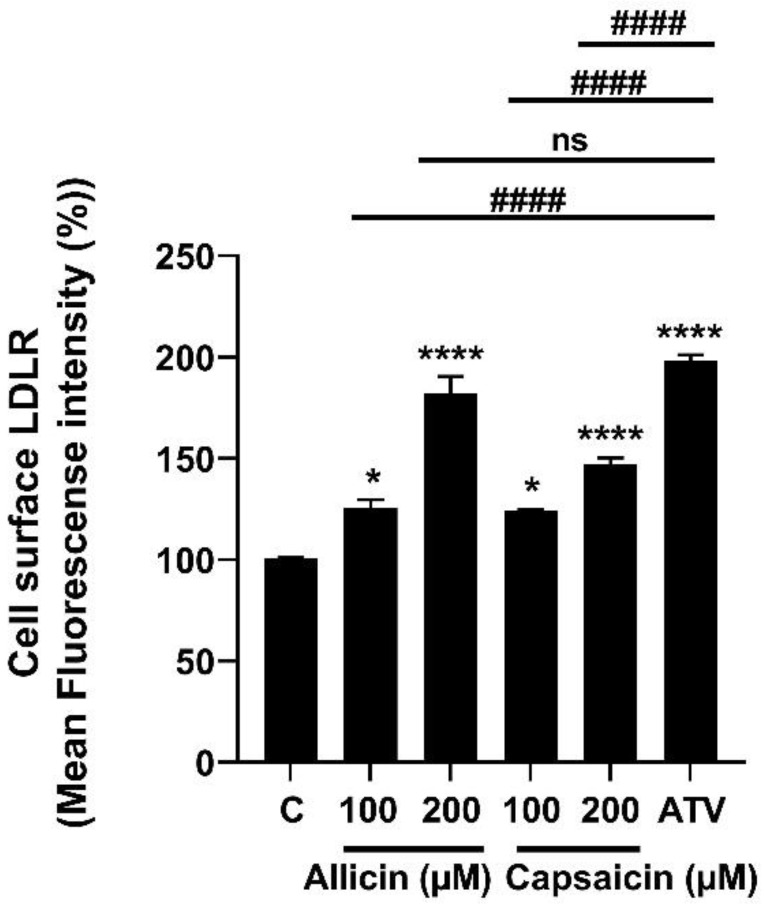
Effects of allicin (100 µM and 200 µM), capsaicin (100 µM and 200 µM), and atorvastatin (10 µM) on LDLR expression in HepG2 cells. HepG2 cells were treated with allicin (100, 200 µM), capsaicin (100 µM, and 200 µM), or atorvastatin (10 µM) for 24 h, and the level of cell-surface LDLR expression was measured by flow cytometric analysis. The data represent the mean ± SEM of three independent experiments (*N* = 3). * *p* < 0.05, and **** *p* < 0.001, represent significant differences compared to the vehicle-treated cells or the control group (C). ^####^ *p* < 0.001 represents significant differences compared to atorvastatin (ATV)-treated cells. ns: not significant.

To investigate the effects of allicin and capsaicin on the level of cell-surface LDL receptor (LDLR), LDLR expression was analyzed. The results showed that allicin (200 µM) significantly increased the expression levels of LDLR mRNA (Figure 2A), whereas capsaicin had no significant effects on the expression levels of LDLR mRNA (Figure 2B). However, allicin (200 µM) and capsaicin (200 µM) significantly increased the expression levels of LDLR protein by approximately 1.33 (*p* < 0.05)-fold and 1.86 (*p* < 0.05)-fold, respectively, compared with vehicle-treated cells or the control group (Figure 3A,B). In addition, effects of allicin and capsaicin on the expression of LDLR on the cell surface were quantitated by flow cytometry analysis. Allicin (200 µM) and capsaicin (200 µM) significantly increased the amounts of cell-surface LDLR on HepG2 cells by approximately 182.33% (*p* < 0.001) and 147.22% (*p* < 0.001), respectively, compared to the control group (Figure 4). The levels of LDLR protein increased by allicin (200 µM) and capsaicin (200 µM) were not significantly different compared with a positive control, atorvastatin (10 µM) (Figure 3A,B).

**Figure 5:**
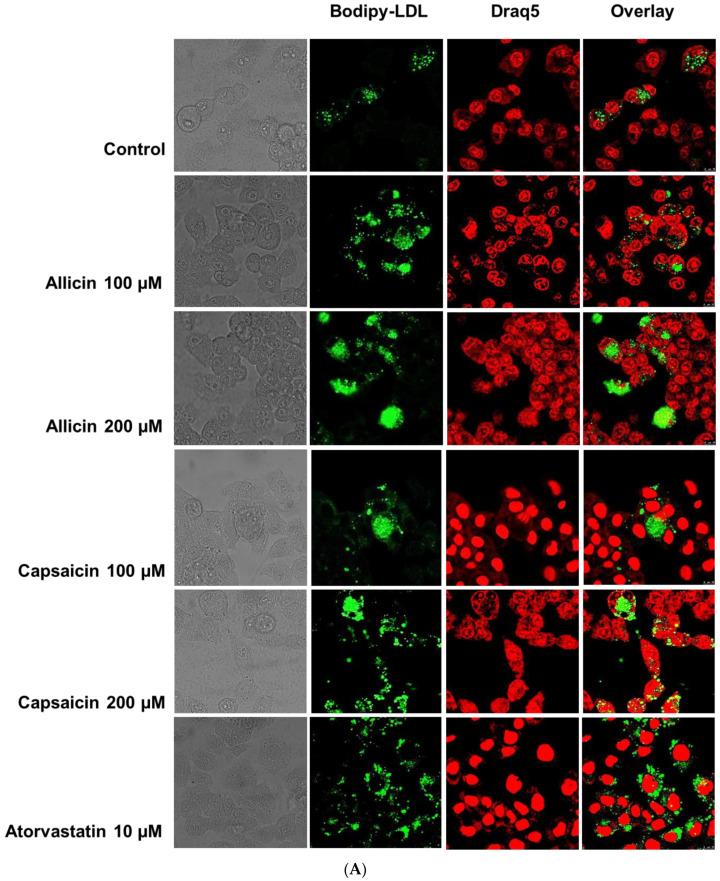

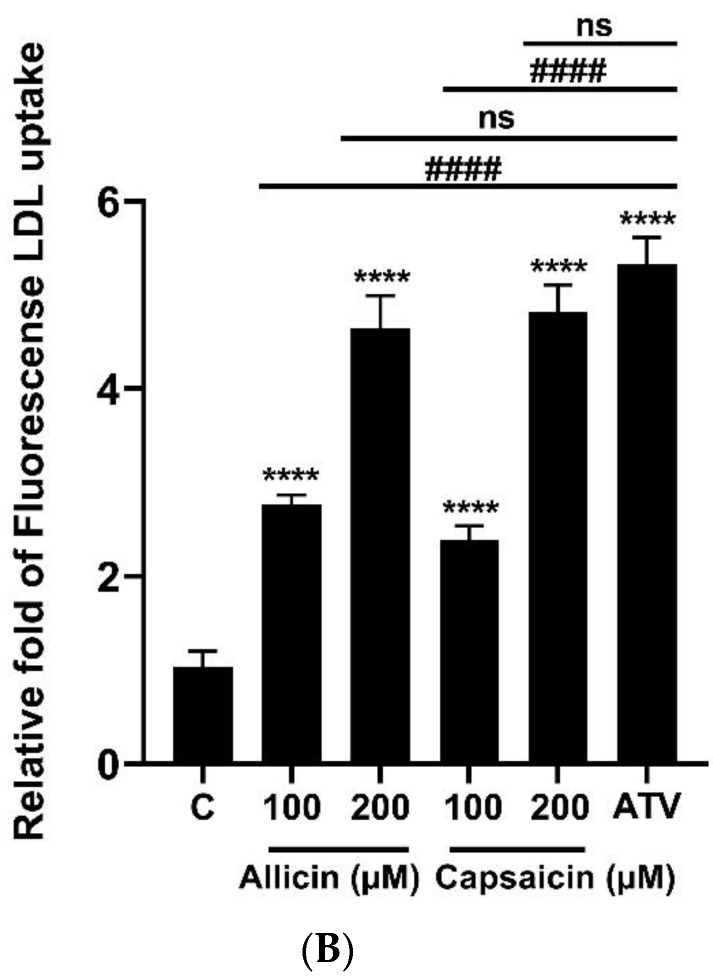
Effects of allicin (100 µM and 200 µM) and capsaicin (100 µM and 200 µM) on LDL uptake in HepG2 cells (**A**). Confocal laser images represent the cell-associated BODIPY-LDL (green), Draq5 (red) and the overlay. A representative blot is shown (**B**). HepG2 cells were treated with allicin (100 µM and 200 µM), capsaicin (100 and 200 µM), or atorvastatin (10 µM) for 24 h, and LDL uptake was determined by confocal laser scanning microscopy. The relative fluorescence intensity was analyzed by ImageJ software. The data represent the mean ± SEM of three independent experiments (*N* = 3). **** *p* < 0.001 represent significant differences compared to the vehicle-treated cells or the control group (C). ^####^ *p* < 0.001 represents significant differences compared to atorvastatin (ATV)-treated cells. ns: not significant.

**Figure 6:**
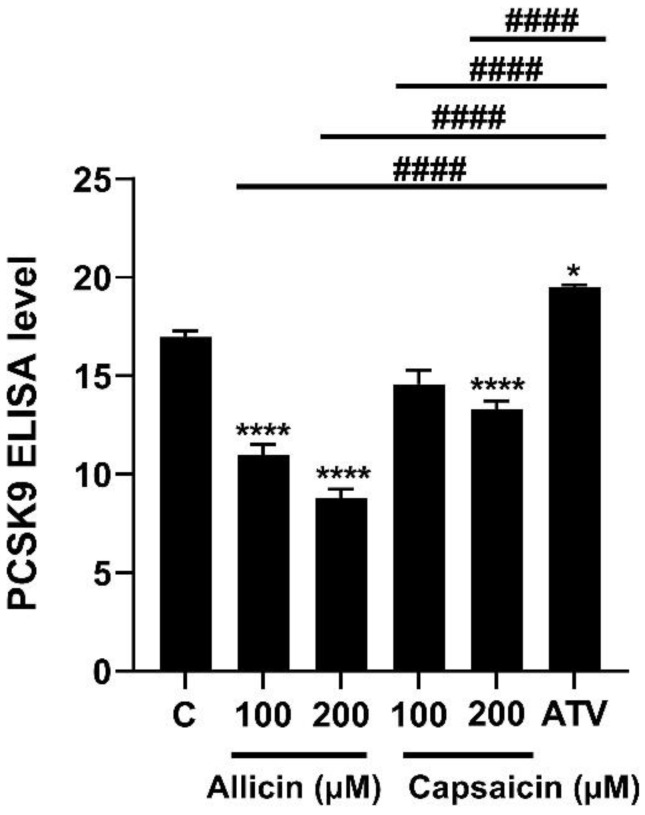
Effects of allicin (100, 200 µM), capsaicin (100, 200 µM), and atorvastatin (10 µM) on PCSK9 expression in HepG2 cells. The PCSK9 levels in culture medium were determined by ELISA. The data represent the mean ± SEM of three independent experiments (*N* = 3). * *p* < 0.05, and **** *p* < 0.001 represent significant differences compared to the vehicle-treated cells or the control group (C); ^####^ *p* < 0.001 represent significant differences compared to atorvastatin (ATV)-treated cells. ns: not significant.

Because LDLR plays a functional role in binding to LDL, LDL is taken up into liver cells [20]. We further investigated the effects of allicin and capsaicin on LDLR activity and LDL uptake in HepG2 cells by confocal laser scanning microscopy (CLSM) (Figure 5A). The results showed that allicin (200 µM) and capsaicin (200 µM) significantly increased LDL uptake into HepG2 cells by approximately 4.64- (*p* < 0.001) and 4.82-fold (*p* < 0.001), respectively, compared to the control group (Figure 5B). These results suggest that allicin and capsaicin increased LDLR protein levels and consequently led to increased LDL uptake into HepG2 cells. The levels of LDL uptake were not significantly different between the allicin (200 µM)- and capsaicin (200 µM)-treated cells or the atorvastatin (10 µM)-treated cells. This indicated that allicin and capsaicin may have similar lipid-lowering potential to atorvastatin. Furthermore, allicin (200 µM) significantly decreased the mRNA expression levels of PCSK9 (Figure 2C), whereas capsaicin had no significant effects on the mRNA levels of PCSK9 in HepG2 cells (Figure 2D). Nevertheless, allicin and capsaicin significantly decreased the protein expression of PCSK9 (Figure 3C,D), as well as PCSK9 levels in culture media (Figure 6). In contrast, atorvastatin (10 µM), which was used as the positive control, significantly increased the mRNA and protein expression levels of PCSK9 (Figure 2C,D and Figure 3C,D).

### 2.3. Effects of Allicin and Capsaicin on SREBP2 and HNF1A Expression in HepG2 Cells, as Well as the Secreted PCSK9 Levels in Culture Media

It is well known that the expression of the LDLR and PCSK9 genes is regulated by SREBP2 [18]. The expression of the PCSK9 gene is also regulated by HNF1α [20]. We further investigated the effects of allicin and capsaicin on the expression of the transcription factors SREBP2 and HNF1α. Although allicin and capsaicin did not significantly change the mRNA levels of SREBP2 (Figure 2E,F), allicin (200 µM) and capsaicin (200 µM) significantly increased the protein expression levels of SREBP2 by approximately 1.19-fold (*p* < 0.01) and 1.28-fold (*p* < 0.05), respectively, compared with the control group (Figure 3E,F). Moreover, capsaicin but not allicin significantly decreased the mRNA expression levels of HNF1α compared with vehicle-treated cells or the control group (Figure 2G,H). In addition, allicin (200 µM) and capsaicin (200 µM) significantly decreased the protein expression levels of HNF1α compared with vehicle-treated cells or the control group (Figure 3G,H). A positive control, atorvastatin (10 µM), significantly increased the mRNA and protein expression levels of SREBP2 and HNF1α (Figure 2E,H and Figure 3E,H).

## 3. Discussion

**Figure 7:**
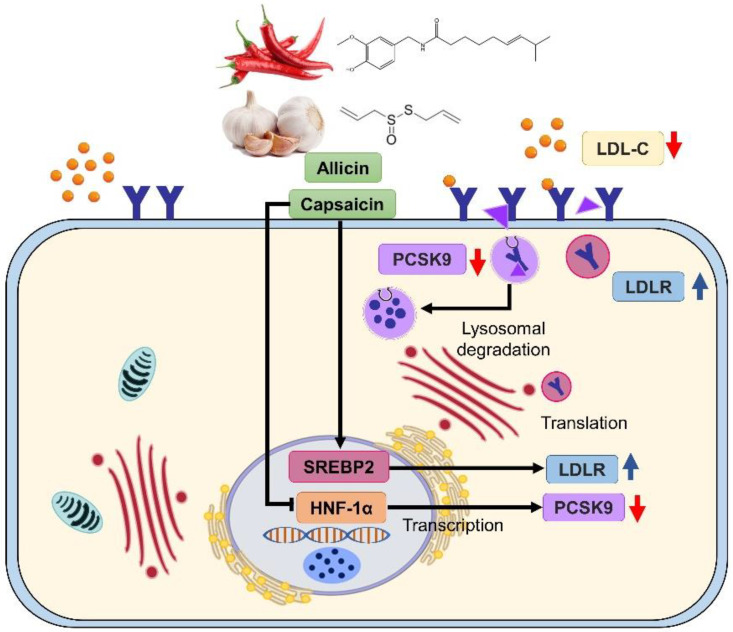
The proposed mechanism of allicin and capsaicin on the hypolipidemic effect in HepG2 cells.

In this study, we demonstrated that allicin and capsaicin possessed hypolipidemic effects by upregulating LDLR expression via SREBP2 activation and downregulating PCSK9 expression through the suppression of HNF1α. In addition, the secreted PCSK9 levels in culture medium were also decreased and may further enhance LDL uptake into HepG2 cells (Figure 7). Our findings were also similar to the previous studies showing that soy β-conglycinin peptides [21], bioactive peptides from hemp seed (*Cannabis sativa*) [22], berberine [23], and lunasin [24] had hypolipidemic effects through decreased PCSK9 expression via the downregulation of HNF1α.

SREBP2 is a transcription factor that regulates the expression of the LDLR and PCSK9 genes [20,25,26]. When intracellular cholesterol is low, SREBP2 is activated and transferred from the endoplasmic reticulum (ER) to the Golgi complex, where it is cleaved into mature SREBP2 [26,27,28]. Mature SREBP2 then enters the nucleus and binds to the promoter region to activate the transcription of the LDLR and PCSK9 genes [29]. Apart from SREBP2, PCSK9 expression is also regulated by another transcription factor, HNF1α [20]. HNF1α binds to the SRE and Sp1 sites on the promoter and turns on the expression of the PCSK9 gene [23]. A previous study showed that HNF1α is a major transcription factor that regulates PCSK9 gene expression in HepG2 cells [20,23].

LDLR and PCSK9 play important roles in cholesterol homeostasis [15,16,20]. LDLR is mostly expressed on liver cells. LDLR binds to apoB100- and apoE-containing lipoproteins, subsequently decreasing the LDL-C in circulation [16]. PCSK9 binds to the EGF-A domain of LDLR and promotes the degradation of LDLR in lysosomes [15,16]. A previous study showed that statins increased PCSK9 levels via the upregulation of SREBP2 [20,30]. This phenomenon resulted in the limitation in the lipid-lowering efficacy of statins [20,30]. Consequently, the PCSK9 inhibitors alirocumab and evolocumab have been developed and are currently used to treat severe hypercholesterolemia patients [31]. Several studies have also discovered natural PCSK9 inhibitors from various medicinal plants or nutrients to further develop lipid-lowering supplements [32]. Thus, we suggest that allicin, capsaicin, and other natural PCSK9 inhibitors be used as cholesterol-lowering agents in hypercholesterolemia subjects or added to statin therapy or other lipid-lowering therapies.

Garlic consumption was shown to reduce serum lipid content in animal and human studies [33,34]. This might be because garlic consists of several active ingredients, such as allicin, ajoene, and DADS [35,36,37,38,39]. Previous studies demonstrated that allicin, ajoene, and water-soluble garlic extracts inhibited sterol biosynthesis [35,36]. Fresh garlic extract and 16 water- or lipid-soluble compounds inhibited squalene monooxygenase and HMG-CoA (3-hydroxy-3-methylglutaryl-coenzyme A) reductase [37,38]. Garlic oil and odorous components of garlic decreased cholesterol levels by reducing hepatic 3-hydroxy-3-methylglutaryl-CoA reductase, cholesterol 7 alpha-hydroxylase, fatty acid synthetase, and pentose-phosphate pathway activities in White Leghorn pullets [40] and enhanced bile acid excretion. Fresh garlic extract reduced microsomal triglyceride transfer protein (MTP) mRNA levels in both human hepatoma HepG2 and intestinal carcinoma Caco-2 cells [41]. Allicin reduced the accumulation of triglycerides (TGs) and total cholesterol (TC) by downregulating SREBP-1 and SREBP-2 in 1,3-DCP-treated HepG2 cells [42]. Garlic supplementation showed significantly lower cholesteryl ester transfer protein (CETP) activity in cholesterol-fed rabbits [43]. Furthermore, allicin reduced palmitic acid-induced lipid accumulation in HepG2 cells [44]. Allicin increased the mRNA expression levels of PPARA and FABP6 and decreased the mRNA expression levels of FABP4 and PPARG [44]. Moreover, diallyl disulfide also increased LDLR expression, promoted LDL uptake, and decreased PCSK9 expression via the downregulation of HNF1α [39].

Furthermore, several studies have demonstrated that the active compounds in chili, capsaicinoids, capsaicin, dihydrocapsaicin, and capsiate display hypolipidemic effects through various molecular mechanisms. An in vivo study showed that rats treated with capsaicinoids exhibited reductions in plasma TG, TC, and LDL-C through the downregulation of HMGCR [45]. Capsaicin protected LDL oxidation in vitro and decreased the level of lipid peroxidation in hypercholesterolemic rats [46]. Capsaicin also reduced hepatic lipogenesis through activated AMPK and inhibited the AKT/mTOR pathway [47]. Moreover, dihydrocapsaicin (DHC) decreased atherosclerotic plaque formation in apoE-/- mice by downregulating apoM expression through inhibiting Foxa2 expression and enhancing LXRα expression in HepG2 cells [48]. Capsiate inhibited lipid accumulation, decreased TG and TC, and increased HDL content in palmitic acid-treated HepG2 cells. Capsiate also increased the levels of AMPK phosphorylation and SIRT1 expression in HepG2 cells but decreased the levels of FGF21 [49]. Taking all of these observations into account, we postulated that the activation or phosphorylation of AMPK by capsaicin may possibly lead to decreased PCSK9 expression via increased SIRT1 expression and the activation of FOXO3a. A previous study proposed that AMPK indirectly activated SIRT1 via intracellular NAD^+^ levels [50]. In addition, SIRT1 has been found to stimulate the transcriptional activity of FOXO3a via FOXO3a deacetylation [51]. FoxO3a protein, a forkhead transcription factor, has been reported to repress PCSK9 gene expression. FOXO3a interacted with the insulin response element (IRE) within the PCSK9 promoter and then interrupted the binding capacity of HNF-1α to the PCSK9 promoter [52].

In the present study, the concentrations of allicin (100–200 µM) and capsaicin (100–200 µM) that were administered to HepG2 cells were nontoxic and equivalent to 16–32 mg/kg and 30–60 mg/kg, respectively. In comparison to in vivo studies, allicin (60 mg/kg in 250 g Wistar rats, equivalent to 15 mg/day) [53] and capsaicin (50 mg/kg in 250 g rats, equivalent to 12.5 mg/day) [54] demonstrated significantly decreased serum TC, LDL-C, and TG. Another study also showed that high-dose administration of capsaicin (0.015%, or 180 mg/kg, equivalent to 18 mg/day) in Wistar rats (120 g) significantly decreased TC and LDL + VLDL [55]. Moreover, the consumption of 9.6 mg allicin per day significantly decreased TC by 4.2% and LDL-C by 6.6% in hypercholesterolemic subjects [56]. These results suggest that the consumption of 9.6–15 mg dietary allicin per day and 12.5–18 mg dietary capsaicin per day may alleviate hypercholesterolemia.

Previous studies proposed that daily intake of 4 g or 1–2 cloves of garlic (equal to 10–18 mg allicin) may provide health benefits [57,58]. Additionally, the daily intake of capsaicinoids among Thai and Mexican populations was 25–200 mg/day [59], whereas a lower daily intake of capsaicinoids (1–30 mg) or capsaicin (0.67–20 mg) was observed in the Korean population [60]. Thus, we determined that the concentrations of allicin and capsaicin used to treat HepG2 cells in this study could be achieved by dietary intake. Nevertheless, excessive consumption of allicin and capsaicin should be avoided because they can cause side effects such as headache, nausea, gastrointestinal discomfort, delayed hemostasis, skin rash, dermatitis, rhinitis, bronchospasms, inhibition of supermatogenesis, garlic breath and body odor for allicin [61], as well as nausea, vomiting, abdominal pain, diarrhea, and gastrointestinal discomfort for capsaicin [62].

This study had some limitations. The effect of HNF1α silencing on PCSK9 expression and the effect of allicin and capsaicin on the signaling pathway were not investigated. Moreover, the molecular mechanisms of allicin and capsaicin and their lipid-lowering effects should be further elucidated.

In conclusion, allicin and capsaicin had hypolipidemic effects in HepG2 cells. Allicin and capsaicin upregulated LDLR expression via the SREBP2 pathway and increased LDL uptake into HepG2 cells. Allicin and capsaicin also decreased the protein expression levels of PCSK9 and HNF1α in HepG2 cells, as well as PCSK9 levels in culture media. These results suggest that allicin and capsaicin can be used as potential supplements for ameliorating hypercholesterolemia.

## 4. Materials and Methods

### 4.1. Chemicals

Allicin, capsaicin, dimethyl sulfoxide (DMSO), 3-(4,5 dimethylthiazol-2-yl)-2,5-diphenyltetrazolium bromide (MTT), and other chemicals were purchased from Sigma-Aldrich Co. (St. Louis, MO, USA). Gibco fetal bovine serum (FBS) and BODIPY-LDL were purchased from Thermo Fisher Scientific, Inc. (Rockford, IL, USA).

### 4.2. Cell Culture and Treatment

The human hepatoma cell line HepG2 was purchased from the American Type Culture Collection (ATCC, USA). Cells were grown in Dulbecco’s modified Eagle’s medium (DMEM, Gibco, Grand Island, NY, USA) supplemented with 10% fetal bovine serum (FBS) (Gibco, Grand Island, NY, USA) and 1% penicillin–streptomycin at 37 °C in 5% CO_2_. HepG2 cells were cultured in serum-starved medium for 24 h and then treated with vehicle (0.1% DMSO), allicin (100 and 200 μM), and capsaicin (100 and 200 μM) for 24 h.

### 4.3. Cell Viability Assay

Cell viability was determined using the MTT assay. Briefly, HepG2 cells were seeded in 96-well plates (1 × 10^4^ cells/well), serum starved for 24 h, and then exposed to different concentrations of allicin (0, 25, 50, 100, and 200 µM) and capsaicin (0, 25, 50, 100, and 200 µM) for 24 h at 37 °C in a humidified atmosphere containing 5% CO_2_. The optical density (OD) value of each well was measured at a wavelength of 560 nm using a microplate reader.

### 4.4. Trypan Blue Exclusion Assay

HepG2 cells were grown in triplicate in 24-well plates (1 × 10^5^ cells/well) in DMEM containing 0.5% FBS for 24 h. Then, the cells were treated with vehicle (0.1% DMSO), allicin (0, 25, 50, 100, and 200 µM), and capsaicin (0, 25, 50, 100, and 200 µM) for 24 h at 37 °C and 5% CO_2_. The cells were trypsinized and centrifuged at 1200× *g* for 5 min. The cell pellet was resuspended in 1 mL of phosphate-buffered saline (PBS). Ten microliters of cell suspension were stained with 10 µL of 0.4% trypan blue (Sigma-Aldrich, UK) for 3 min. The viable, unstained cells were counted in 3 different fields with a threshold value of 200 cells per field using a hemocytometer within 5 min.

### 4.5. Flow Cytometric Analysis of Cell-Surface LDLR

HepG2 cells were seeded at 1 × 10^6^ cells/well in 6-well plates. Briefly, cells were seeded as described above and were then incubated with DMEM containing 0.5% FBS for 24 h. After that, cells were treated with vehicle, allicin (100 and 200 μM), or capsaicin (100 and 200 μM) for an additional 24 h. Cells were detached by adding 1 mL/well cell dissociation solution (CDS), washed with PBS, and incubated with PBS containing 1% bovine serum albumin at room temperature for 30 min. After blocking, the cells were incubated with the anti-LDLR antibody (1:400) at room temperature for 1 h, washed with PBS (×2), and then incubated with Alexa Fluor 488-conjugated goat anti-rabbit IgG (1:1000) (Thermo Fisher Scientific) at room temperature for 1 h. Cells were resuspended in PBS and analyzed by flow cytometry (FACScan, BD Biosciences, San Jose, CA, USA). Data were calculated using Cell Quest Pro software (BD Biosciences), and the cell surface LDLR was expressed as the relative percentage of the geometric mean fluorescence intensity (MFI).

### 4.6. BODIPY-LDL Uptake Assay

HepG2 cells were seeded in 6-well plates. Following treatment with various concentrations of allicin (0, 100, and 200 µM) and capsaicin (0, 100, and 200 µM) for 24 h, the cells were rinsed twice with PBS. Subsequently, the cells were incubated with 5 μg/mL BODIPY^TM^-FL-LDL (Thermo Fisher Scientific) for 4 h at 37 °C, followed by staining with 5 µM Draq5 (Thermo Fisher Scientific) for 15 min at 37 °C. The cells were fixed in 4% paraformaldehyde for 15 min at room temperature. Immunofluorescence was visualized using confocal laser scanning microscopy (CLSM).

### 4.7. Reverse Transcription Quantitative PCR (RT-qPCR) Analysis

Cells were seeded as described above and treated with vehicle, allicin, and capsaicin for 24 h. RNA was extracted from cells using the Total RNA Mini Kit (Geneaid, Taipei, Taiwan). Reverse transcription was performed using the iScript™ Reverse Transcription Supermix for RT-qPCR (Bio-Rad). Quantitative real-time PCR was performed using a reaction mixture containing cDNA, primers (Table 1) [63,64,65], and Luna^®®^ Universal qPCR Master Mix (New England Biolabs, Inc.). PCR amplification was performed using the Applied Biosystems QuantStudio 5 Real-Time PCR System (Applied Biosystems, Massachusetts, United States). The relative differences in expression between groups were analyzed using the ΔΔCt method and normalized to the GAPDH levels in the same samples.

### 4.8. Western Blot Analysis

Cells were seeded as described above and treated with vehicle, allicin (100, 200 µM), or capsaicin (100, 200 µM) for 24 h. Total cellular proteins were harvested using cell lysis buffer (Thermo Fisher Scientific, Inc., Rockford, IL, USA). The protein samples were separated by 10% SDS−PAGE and transferred to nitrocellulose membranes (PerkinElmer, Boston, MA, USA). The membranes were incubated with the following specific antibodies: anti-LDLR (ab204941), anti-PCSK9 (ab84041), anti-SREBP2 (ab194667), anti-β-actin (ab8226), and anti-HNF1α (Thermo Fisher). The membranes were incubated with HRP-conjugated goat anti-mouse IgG secondary antibody (ab205719), and the proteins were detected using Clarity Western ECL Substrate (Bio-Rad). The chemiluminescent signal was visualized using a ChemiDoc Imaging System (Bio-Rad).

### 4.9. Enzyme-Linked Immunosorbent Assay Analysis

HepG2 cells (5 × 10^5^ cells/well) were seeded into a 6-well plate and were grown for 24 h in complete medium; then, the cells were washed twice with PBS and starved in DMEM supplemented with 0.5% FBS for 24 h. Next, the cells were washed twice with PBS and treated with allicin (100, 200 µM), capsaicin (100, 200 µM), or atorvastatin in DMEM supplemented with 0.5% FBS for 24 h; finally, the cell culture media were collected. Then, the secreted PCSK9 in the media was measured using a PCSK9 ELISA kit (Biolegend) according to the manufacturer’s instructions.

### 4.10. Statistical Analyses

All experiments were performed at least three times. Statistical analyses were performed using SPSS 23.0 software (SPSS Inc., Chicago, IL, USA). Bar graphs were plotted using GraphPad Prism version 9.0 (GraphPad software LLC, San Diego, CA, USA). After the Shapiro–Wilk normality test, the results were analyzed using one-way ANOVA followed by the Bonferroni test for parametric factors or the Kruskal Wallis, and Mann–Whitney U test for nonparametric factors. A *p* value of <0.05 was considered statistically significant.

## Figures and Tables

**Table 1:** Primer sequences used for RT-qPCR.

Gene Name	Primer Sequence
LDLR	FP: 5′-AGTTGGCTGCGTTAATGTGA-3′
	RP: 5′-TGATGGGTTCATCTGACCAGT-3′
PCSK9	FP: 5′-GCTGAGCTGCTCCAGTTTCT-3′
	RP: 5′-AATGGCGTAGACACCCTCAC-3′
SREBP2	FP: 5′-CTCTGACCAGCACCCACACT-3′
	RP: 5′-CACACCATTTACCAGCCATAAG-3′
HNF1α	FP: 5′ TGGCGCAGCAGTTCACCCAT 3′
	RP: 5′ TGAAACGGTTCCTCCGCCCC 3′
GAPDH	FP: 5′-CATGAGAAGTATGACAACAGCCT-3′
	RP: 5′-AGTCCTTCCACGATACCAAAGT-3′

## Data Availability

The data presented in this study are available on request from the corresponding author.
